# A conserved RNA structure at the capsid-coding sequence of Zika virus genome is required for viral replication in a host-dependent manner

**DOI:** 10.1128/jvi.01550-25

**Published:** 2025-10-13

**Authors:** Guadalupe S. Costa Navarro, Horacio M. Pallarés, María Mora González López Ledesma, Luana de Borba, Romina Mazzolenis, Andrea V. Gamarnik

**Affiliations:** 1 Fundación Instituto Leloir-CONICET 62873 , Buenos Aires, Argentina; Iowa State University, Ames, Iowa, USA

**Keywords:** Zika virus, cis-acting viral RNAs, flavivirus, viral genome cyclization, viral RNA, RNA structures in coding sequences

## Abstract

Flaviviruses are emerging and re-emerging pathogens causing widespread epidemics worldwide. Their RNA genomes play multiple roles during infection, folding into dynamic structures that regulate viral processes. To understand the mechanisms of flavivirus infection and to design genetic tools for viral countermeasures, it is important to dissect functional RNA structures present in viral genomes. Here, we investigate RNA structures within the open reading frame of the Zika virus (ZIKV) genome that regulate viral replication. We identified a functional stem-loop structure, SL1, located within the conserved C1 element in the capsid protein coding sequence of mosquito-borne flavivirus genomes. The integrity of the SL1 structure was crucial for viral RNA amplification in mosquito cells and enhanced ZIKV replication in vertebrate cells. Evolution experiments in mosquito cells with lethal SL1-disrupting mutants revealed reversions and pseudo-reversions that restored SL1 structure, confirming its role as a cis-acting RNA element. We also found that a sequence within SL1 contributes to a novel genome cyclization element unique to ZIKV. This sequence folds locally into SL1 or hybridizes with a 3’ UTR sequence to extend the conserved cyclization sequence (CS1), which is known to be essential for RNA synthesis. Although the C1 element is conserved among mosquito-borne flaviviruses, the RNA structures and long-range interactions in this element required for ZIKV replication differ from those reported for dengue virus. Our studies highlight the presence of a conserved RNA element operating through distinct mechanisms in related flaviviruses. These findings offer insights into the dynamic nature of the ZIKV genome and provide information for rational flavivirus attenuation.

**IMPORTANCE:**

Flaviviruses are important human pathogens mainly vectored by arthropods. They contain RNA genomes that fold into complex structures with biological functions in viral infection. Zika virus is a flavivirus that has caused significant outbreaks and epidemics around the world. In this study, we used Zika virus to identify functional RNA structures present in the viral coding sequence. We manipulated an infectious clone from an Argentinean Zika virus isolate to dissociate protein-coding sequences from cis-acting RNA structures and discovered an RNA element in the capsid coding region that is essential for Zika virus replication in mosquito cells. Point mutations, disrupting the identified structure, impaired infection in mosquito cells and rendered viral attenuation in mammalian cells. Selection of revertant viruses in cell culture restored the RNA structure and the viral replication capacity. Our studies provide a basic understanding of the flavivirus genome organization, which is necessary for designing rational antiviral strategies.

## INTRODUCTION

Viral RNA genomes play a myriad of functions during infection. In addition to coding for viral proteins, these molecules contain RNA regulatory elements in the coding and non-coding regions that govern viral replication and participate in processes to escape or trigger host antiviral responses (1–4). A combination of RNA structure evolutionary conservation studies, high-throughput RNA structure mapping, and functional analysis highlights the complexity of the spatial organization of viral RNAs (5–8). RNA folding includes local and long-range RNA-RNA interactions that determine the overall organization of viral genomes (9). Viral RNAs exist as dynamic ensembles including multiple conformations (6, 10). The composition of these ensembles depends on genome engagement with the translation or RNA replication machinery, the cell environment, and the interaction with viral and cellular proteins. For instance, an equilibrium between different conformations of flavivirus genomes is crucial for viral RNA synthesis (11, 12). In this regard, RNA structures with alternative conformations were found to be essential for viral replication, indicating the flexibility and the multiple folding properties of RNA genomes in the infected cell (11, 13).

Recent studies have provided important information regarding RNA genome folding using global mapping of RNA-RNA interactions in different pathogenic viruses (6–8, 14). The challenge is to define their roles and mechanisms involved in viral infection. Here, we used the Zika virus (ZIKV) to assess the relevance of RNA structures present in the viral coding sequence. ZIKV is a mosquito-borne flavivirus that emerged in 2015 as a pathogen of global concern that spread across South and Central America (15, 16). The flavivirus genus includes a number of important human pathogens such as dengue virus (DENV), West Nile virus (WNV), Japanese encephalitis virus (JEV), tick-borne encephalitis virus (TBEV), and yellow fever virus (YFV) (17). Flaviviruses contain a positive-strand RNA genome of about 11 kb, encoding at least ten viral proteins in a single open reading frame. A number of conserved RNA structures present in the coding and non-coding regions of flavivirus genomes have been identified to modulate RNA replication and counteract antiviral responses (13, 18–24). In this regard, flavivirus replication requires long-range RNA-RNA interactions that lead to genome cyclization, a conformation that is essential for polymerase initiation during viral RNA synthesis (12). Long-range interactions are mediated by complementary sequences, including the conserved sequence 1 (CS1) (25), the upstream AUG region (UAR) (26, 27), the downstream AUG region (DAR) (28, 29), and other sequences that are virus-specific. Also, RNA structures at the 3’UTR of flavivirus genomes participate in both RNA replication and immune evasion mechanisms. Complex folding of RNA structures, known as xrRNAs, inhibits 5’ to 3’ degradation of the viral genome (30, 31). This leads to the production of non-coding RNAs, sfRNAs, which accumulate during infection and counteract host antiviral responses. Recent data on the mechanistic aspects of how ZIKV sfRNA evades immune responses have been reported (32, 33).

RNA structures that modulate flavivirus replication have also been described in protein-coding sequences. Downstream of the cyclization elements in the coding sequence of the capsid protein, the conserved capsid-coding region hairpin (cHP) has been proposed to modulate translation initiation (34) and to be essential for RNA replication in DENV and WNV (22); however, the mechanism by which the cHP participates in viral RNA replication is still unclear. More recently, using DENV as a model, an additional structure downstream of the cHP, named C1 or DCS-PK, has been described to enhance viral replication by modulating genome cyclization (20, 35).

Flaviviruses naturally alternate between mosquitoes and humans, replicating efficiently in these two very different hosts. In this regard, there are flavivirus RNA structures that are essential for infection in both hosts, whereas there are RNA elements that play host-specific functions (3, 36–38). Adaptation of flaviviruses to a specific host often results in the emergence of viral variants that replicate more efficiently in that host, at the expense of reduced fitness in the alternative host. Some of these adaptive variations involve alterations in viral RNA structures, highlighting a trade-off where the RNA adopts a suboptimal folding state that balances the requirements of both hosts without being fully optimized for either. For example, we have previously demonstrated in DENV that restricting viral replication in mosquitoes or mosquito cells leads to the accumulation of mutations in the 3’ UTR of the viral genome. These mutations enhance replication in mosquitoes but diminish infection in human cells (13, 39).

Here, we identified a stem-loop structure, SL1, located within the coding sequence of the capsid protein, as essential for ZIKV infection in mosquito cells while having a minor impact on replication in mammalian cells. SL1 is part of the C1 structure (also referred to as DCS-PK), previously described as relevant for DENV infection. However, our findings reveal that the functional elements within C1 differ between ZIKV and DENV. Mutations that disrupt and reconstitute the SL1 structure in ZIKV, without altering the encoded protein, demonstrate the critical role of SL1 in viral RNA replication. In addition, lethal mutations targeting SL1 produced revertant and pseudo-revertant viruses that restored the structure, indicating strong selective pressure to maintain its integrity. In DENV2, C1 interacts with a conserved 3’UTR structure known as DB1, which is absent in the ZIKV genome. In this study, we found that a sequence within SL1 is complementary to a sequence at the viral 3’UTR present upstream of 3’CS1. Mutational analysis and reconstitution of this potential long-range interaction confirmed its requirement for ZIKV RNA synthesis in a host-dependent manner. Together, these findings provide new insights into the functional RNA elements of the ZIKV genome and underscore the distinct roles of conserved RNA structures in closely related flaviviruses.

## RESULTS

**Fig 1:**
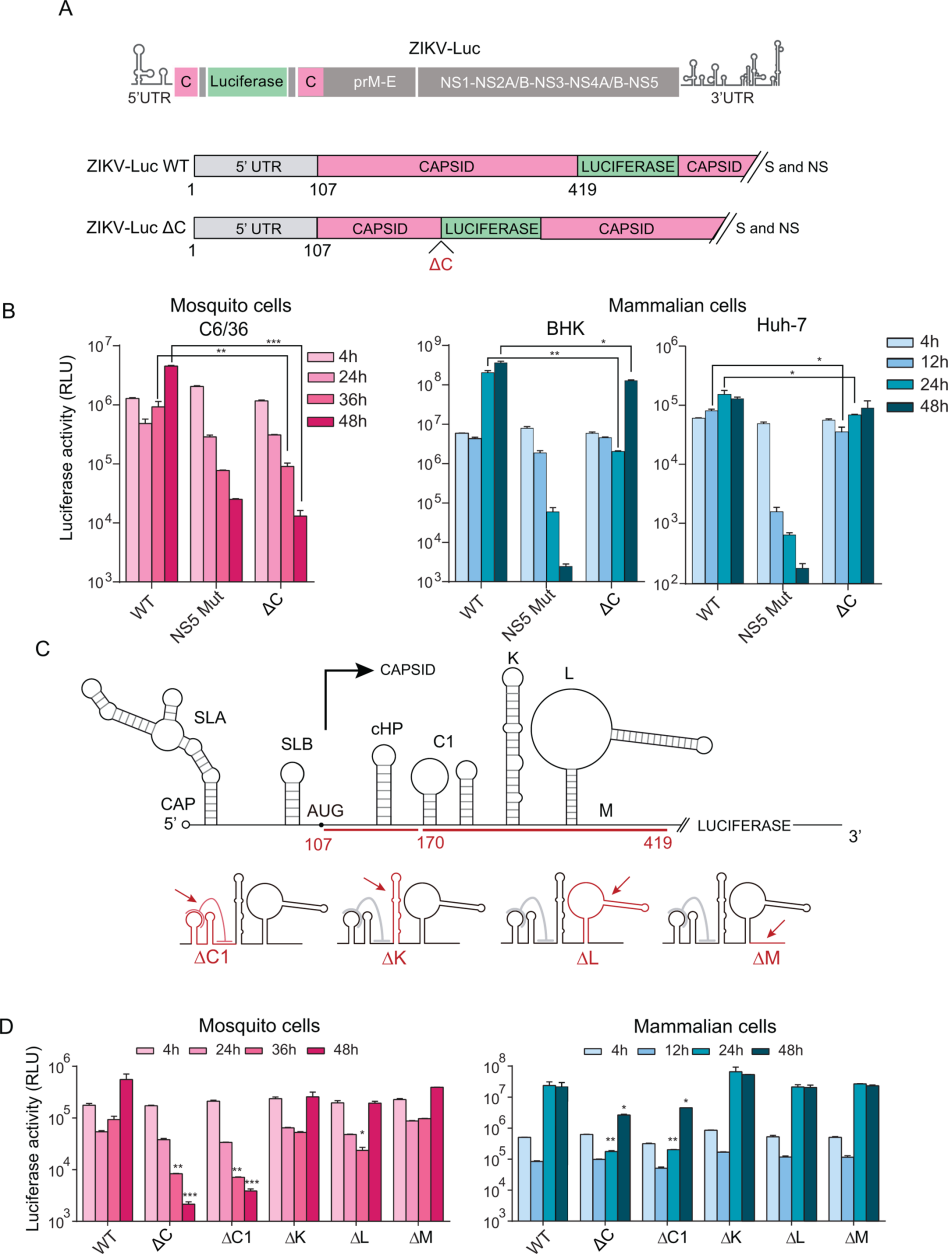
Cis-acting RNA structures in the ZIKV capsid-coding region are essential for viral replication in mosquito cells. (**A**) Schematic representation of the reporter ZIKV-Luc, showing the location of the insertion of the luciferase gene and the duplicated copy of the capsid-coding sequence. The ZIKV-Luc WT and the mutant ΔC are shown. (**B**) ZIKV-Luc RNA replication in transfected mosquito and mammalian cells. Luciferase activity levels of ZIKV-Luc WT, ZIKV-Luc ΔC, and the replication-impaired NS5 Mut are shown as a function of time (h: hours post-transfection) for mosquito C6/36, BHK, and human Huh-7 cells. (**C**) Top, schematic representation of the predicted structures within the 5’ UTR and the capsid-coding region of ZIKV. Relevant RNA elements are indicated: stem-loop A (SLA), stem-loop B (SLB), translation initiator AUG, c-Hairpin (cHP), and C1, K, and L structures. M represents the unstructured 3’ region of the capsid-coding sequence. Bottom, representation of deletion mutants of C1 (ΔC1), K (ΔK), and L (ΔL) structures, and the M sequence (ΔM). Deletions are represented in red and indicated with arrows. (**D**) Replication of reporter ZIKV WT and mutants in transfected mosquito or mammalian cells as a function of time (h: hours post-transfection). The luciferase expression shown corresponds to one representative experiment from three biological replicates in each case. Graphs display the mean ± standard deviation (SD) of two technical replicates per condition. Pairwise comparisons between WT and mutant groups at each time point were performed using an unpaired one-tailed Student’s *t*-test. Differences were considered statistically significant at *P* values < 0.05 *, <0.01 **, and <0.001 ***. Statistical analysis was performed using GraphPad Prism software.

Flavivirus infectious clones and reporter viruses have been instrumental tools to dissect functions and mechanisms of viral RNA elements. To identify RNA structures in the ZIKV coding sequence that modulate viral RNA replication, we used an infectious clone constructed from an Argentinean ZIKV clinical isolate (36). In this system, a luciferase gene was included, and the capsid protein coding region was duplicated to dissociate potential cis-acting RNA elements that regulate RNA replication from the protein coding sequence (ZIKV-Luc, Fig. 1A). The capsid sequence immediately following the 5’ UTR was used to interrogate cis-acting RNA elements that could modulate the SLA promoter activity present at the 5’ end of the genome, whereas the second copy of capsid followed by prM-E provided the structural proteins for viral encapsidation. Using this construct, we generated a deletion mutant (ZIKV-Luc ΔC, Fig. 1A), retaining only the first 62 nucleotides of the first capsid-coding sequence, containing the known cyclization sequence 1 (CS1) and the conserved cHP.

Genomic RNA from the ZIKV-Luc WT and ΔC was quantified, and equal amounts of RNA were transfected into mosquito C6/36 and mammalian BHK or Huh-7 cells together with a control that was replication impaired, containing a mutation in the polymerase NS5 catalytic site (NS5 Mut). Luciferase activity was evaluated as a function of time (Fig. 1B). At 4 h post-transfection (hpt), the luciferase activity measured indicates translation of the input RNA. The difference in luciferase activity between WT and NS5 Mut after 24 hpt represents amplification of the viral genome by the viral NS5 polymerase (compare WT and NS5Mut, Fig. 1B). The ZIKV-Luc WT replicates efficiently in mosquito and vertebrate cells (BHK and human Huh-7). The ZIKV-Luc ΔC shows efficient translation of input RNA but fails replication in mosquito cells, displaying luciferase levels comparable with those of the NS5 Mut replication-impaired control (Fig. 1B). In BHK cells, ZIKV-Luc ΔC replicates but produces significantly lower luciferase levels at 24 h compared with the WT. In human Huh-7 cells, ZIKV-Luc ΔC supports efficient translation of the input RNA but shows a slight reduction in RNA amplification at 12 and 24 hpt (Fig. 1B). The data show that deletion of the capsid coding region reduces viral replication in hamster and human cells while abolishing replication in mosquito cells.

To identify functional RNA sequences or structures missing in the ZIKV-Luc ΔC mutant that may be relevant for viral RNA replication, we systematically deleted each of the previously predicted RNA elements in this region (7, 14, 20, 35) (C1, K, L, and M) in the context of ZIKV-Luc (Fig. 1C). Viral RNAs were generated from ZIKV-Luc ΔC1, ΔK, ΔL, and ΔM mutants, and equal amounts of quantified RNA were transfected into cells alongside the WT and the NS5 Mut control. At 4 hpt, luciferase levels for all mutants were comparable to WT, indicating that the transfected RNAs were efficiently translated (Fig. 1D). Reporter viruses with deletions in the K, L, or M structures produced viral RNAs that were successfully amplified in both mosquito and mammalian cells, as evidenced by luciferase levels after 24 hpt. In contrast, deletion of the C1 element impaired viral RNA replication in mosquito cells and significantly delayed RNA amplification in mammalian cells. In both cell types, the phenotypes of ZIKV-Luc ΔC1 were comparable with those observed with the mutant carrying the large deletion, ZIKV-Luc ΔC.

**Fig 2:**
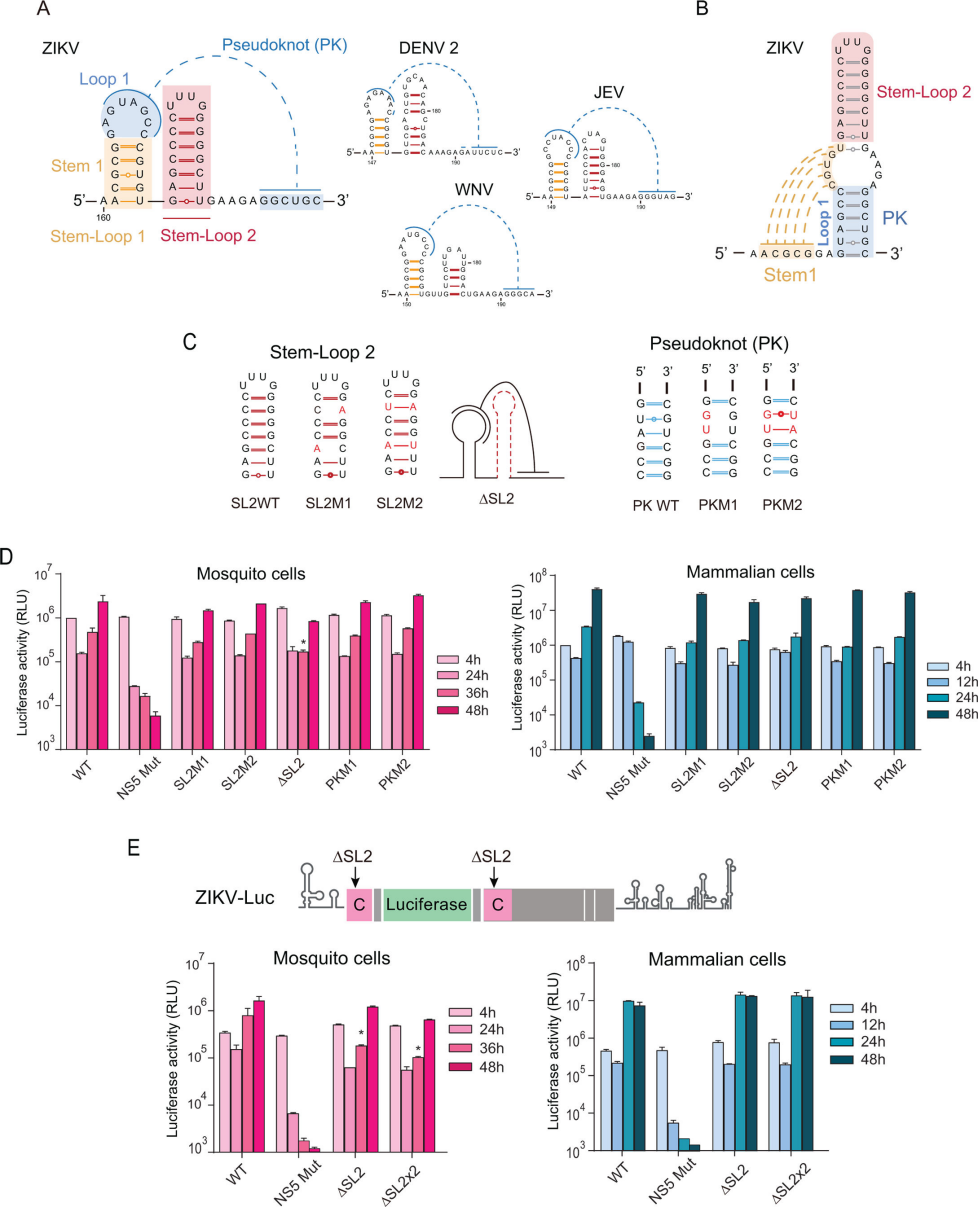
The C1 RNA structure is conserved in different mosquito-borne flaviviruses. (**A**) Elements of the C1 structure: Stem-loop 1, Stem-loop 2, and PK interaction. The C1 structure of ZIKV, DENV, JEV, and WNV is shown. (**B**) Alternative representation of C1 that is equivalent to the one shown in A. (**C**) The mutations within Stem-loop 2 and PK are indicated. (**D**) Luciferase expression levels of ZIKV-Luc WT and mutants are shown as a function of time in both cell types, as indicated (h: hours post-transfection). (**E**) On the top, a schematic representation of the mutants carrying the deletion of one or both copies of SL2. On the bottom, luciferase expression levels of ZIKV-Luc WT and mutants with deletion of one or two copies of SL2 are shown as a function of time in both cell types, as indicated (h: hours post-transfection). The luciferase expression shown corresponds to a representative experiment from three biological replicates in each case. Graphs display the mean ± standard deviation (SD) of two technical replicates per condition. Pairwise comparisons between WT and mutant groups at each time point were performed using an unpaired one-tailed Student’s *t*-test. Differences were considered statistically significant at *P* < 0.05 (*).

The C1 region has been previously shown to be conserved across various mosquito-borne flaviviruses (20). SHAPE analysis performed in our laboratory and others indicates that C1 of DENV folds into a short SL1, a longer SL2, and a pseudoknot (PK) interaction with downstream nucleotides (20, 35) (Fig. 2A). More recently, chemical probing using ZIKV predicted a C1 region (also named DCS-PK) (9), which accommodates the same 2D structure as the one proposed using SHAPE for DENV and other flaviviruses (Fig. 2B). Using IPKnot as a tool for predicting RNA secondary structures that can model pseudoknot interactions, we obtained a C1 structure similar to the one previously proposed (Fig. S1). In addition, we analyzed the secondary structure projection of the 3D prediction of C1 obtained using AlphaFold3, which also predicted the formation of SL1, SL2, and a PK (Fig. S2).

Although it might be expected that the C1 RNA structure serves a similar function in different flaviviruses, its requirement for ZIKV replication was unclear. This is because ZIKV lacks the DB1 structure in the 3’UTR, which contains complementary sequences that enhance genome cyclization through hybridization with the C1 sequence in DENV (13, 35). To address this question, we decided to further investigate the elements of C1 that are required for ZIKV replication.

We first analyzed the role of sub-elements of C1, assessing the function of SL2 and the PK interaction on ZIKV replication. To this end, we generated ZIKV mutants designed to disrupt and reconstitute the SL2 stem (SL2M1 and SL2M2) and created a mutant lacking the entire SL2 structure (ΔSL2). Additionally, we constructed a mutant disrupting the PK complementarity (PKM1) and another reconstituting it via mutations in the complementary strand (PKM2, Fig. 2C). Predictions of the RNA structures of SL2 and PK mutants suggest that the overall C1 was maintained and PKM2 restored PK structure (Fig. S2).

RNAs corresponding to SL2M1, SL2M2, ΔSL2, PKM1, and PKM2, along with WT and NS5 Mut control, were quantified, and equal amounts were transfected into mosquito and mammalian cells. The data show that RNAs from ZIKV-Luc mutants with SL2 disruptions or deletion were able to replicate in both host cell types (Fig. 2D). The ΔSL2 mutant exhibited a slight but significant replication delay compared with the WT reporter ZIKV but ultimately reached near WT levels by 48 hpt. Similarly, transfection of the PKM1 and PKM2 mutant RNAs showed efficient translation and replication in both hosts. These data suggest that the SL2 and the PK do not play a relevant role as cis-acting elements for RNA replication, which differs from that previously reported by our lab and others in DENV (20, 35). It has been previously shown that the SL2 and PK elements of the C1 structure enhance DENV RNA amplification.

To further investigate a possible role of SL2, we examined whether the SL2 structure present in the second copy of the capsid protein could provide a function when the upstream copy was deleted. To accomplish this, we generated a new ZIKV mutant with a deletion of both SL2 copies (Fig. 2E). Viral RNA levels from WT, NS5Mut, and ΔSL2 mutants, either with one or both copies deleted, were transfected, and luciferase activity was measured over time. The results show that the ZIKV-Luc mutant with double SL2 deletion replicates in both cell types, suggesting that this structure does not play a critical role in RNA amplification. It is important to mention that mutants lacking one or both SL2 copies exhibit a slight reduction in luciferase levels at 36 hpt compared with the WT in mosquito cells (Fig. 2E).

**Fig 3:**
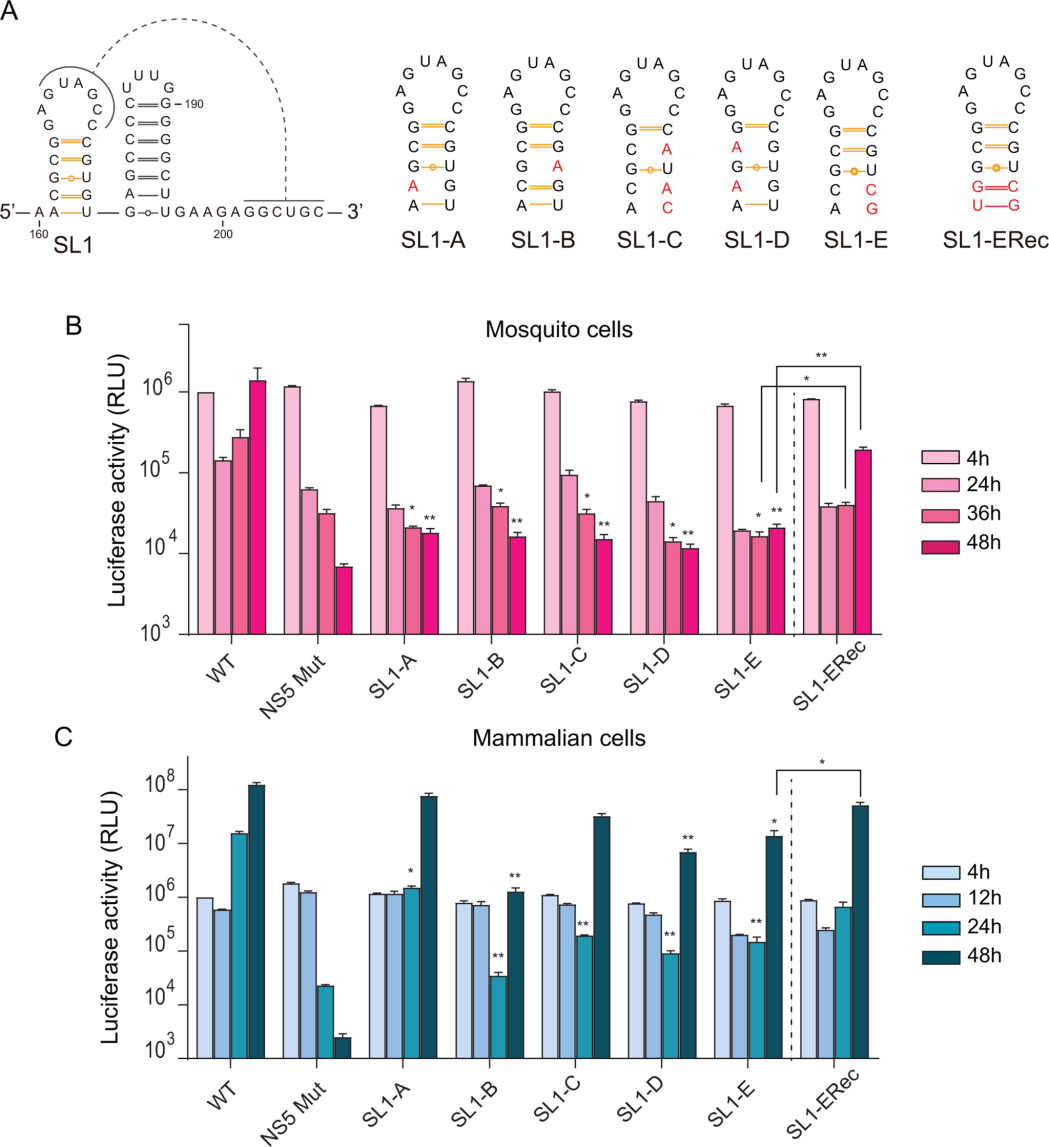
The stem loop 1 (SL1) structure plays an essential role for ZIKV replication in mosquito cells. (**A**) Representation of mutations incorporated within SL1, disrupting and reconstituting the stem structure, in the ZIKV-Luc. (**B**) Luciferase expression levels of ZIKV-Luc WT and mutants are shown as a function of time in mosquito cells (h: hours post-transfection). (**C**) Same as (**B**) in mammalian BHK cells. The luciferase expression shown corresponds to a representative experiment from three biological replicates in each case. Graphs display the mean ± standard deviation (SD) of two technical replicates per condition. Pairwise comparisons between WT and mutant groups at each time point were performed using an unpaired one-tailed Student’s *t*-test. For the reconstitution mutant (SL1-ERec), comparisons were made between SL1-E and SL1-ERec, as indicated in the graph. Differences were considered statistically significant at *P* values < 0.05 * and <0.01 ** . Statistical analysis was performed using GraphPad Prism software.

To investigate the potential role of SL1, we generated a series of ZIKV mutants carrying substitutions on either side of the stem that disrupt the structure (SL1-A, SL1-B, SL1-C, SL1-D, and SL1-E, Fig. 3A). Secondary structure predictions of mutant RNAs were analyzed using IPKnot and AlphaFold3, which indicated that SL2 and the PK were maintained (Fig. S1 and S2). Transfection of the corresponding viral RNAs showed that disrupting SL1 stem abolishes ZIKV replication in mosquito cells while causing a delay in viral replication in mammalian cells (Fig. 3B and C). We also used the mutant SL1-E to introduce additional substitutions to restore base pairing (SL1-ERec). Transfection of ZIKV-Luc SL1-ERec resulted in a significant increase in luciferase with respect to the SL1-E mutant (Fig. 3B). Although the reconstituted mutant did not fully restore WT levels of RNA amplification, the results support the importance of the SL1 structure for ZIKV replication, particularly in mosquito cells.

**Fig 4:**
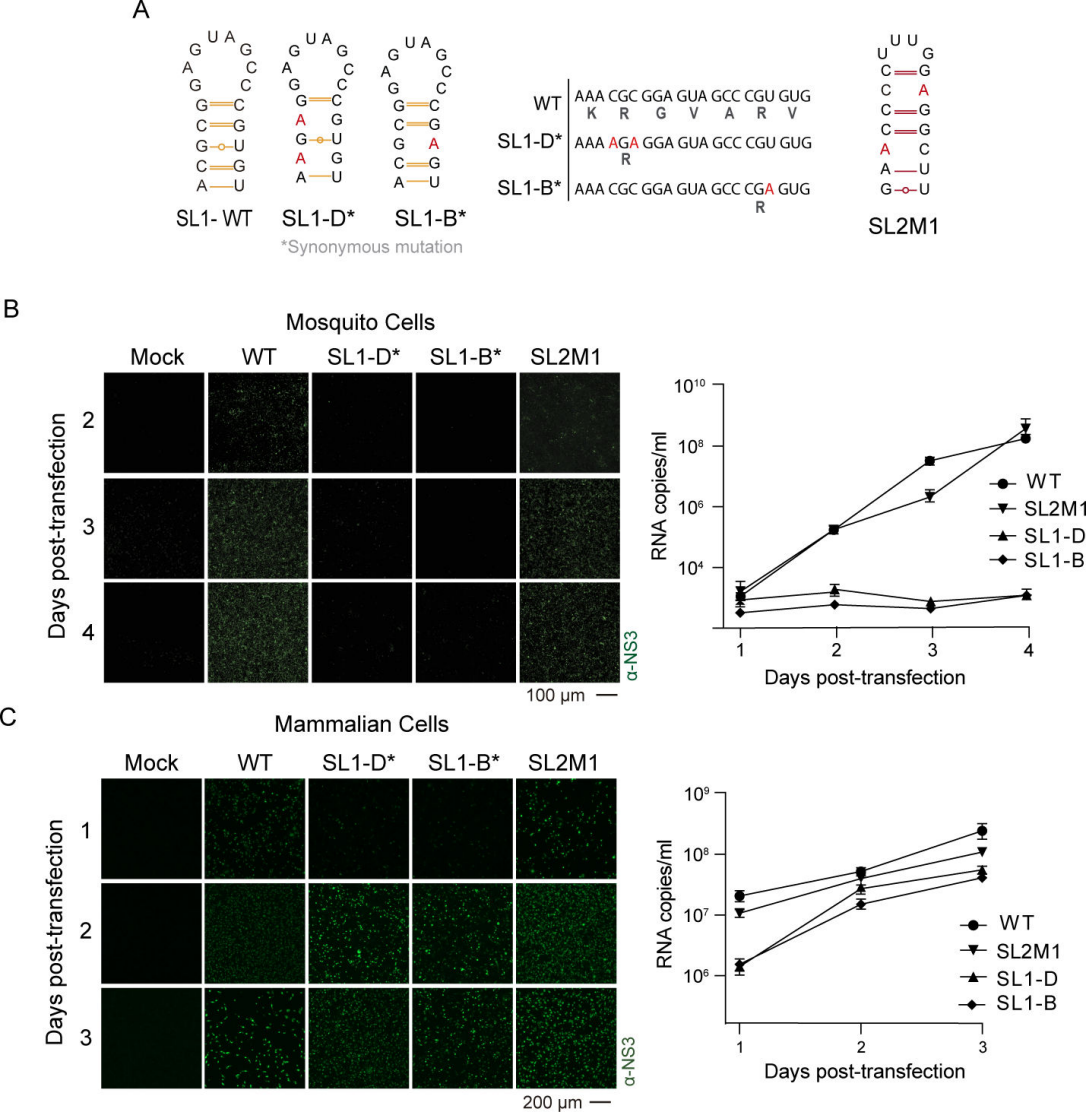
Replication kinetics of ZIKV mutants that altered the SL1 structure by synonymous changes in the capsid coding sequence. (**A**) Representation of synonymous mutations (*) incorporated within SL1 (SL1-D and SL1-B) in the context of the ZIKV infectious clone, indicating the corresponding amino acid sequence of the capsid protein. The sequence of mutant SL2M1 is also shown. (**B**) On the left, an immunofluorescence assay showing the propagation of ZIKV WT and mutants as a function of time in mosquito cells. Infected cells were labeled with specific ZIKV anti-NS3 antibodies. On the right, quantification of viral RNA by RT-PCR from secreted particles harvested in the supernatant of transfected cells, and values are means ± standard deviations. (**C**) Same as (**B**) in mammalian BHK cells.

To further study the role of different elements of C1, instead of using the reporter ZIKV-Luc, we used the ZIKV WT infectious clone that contains a single capsid coding region. In this case, we introduced synonymous substitutions to disrupt SL1 structure, maintaining the capsid protein sequence (SL1-D and SL1-B) and included a mutant disrupting the stem of the SL2 structure (SL2M1) (Fig. 4A). Equal amounts of viral RNAs were transfected into the two host cell types, and viral propagation was evaluated by immunofluorescence assay (IFA) using antibodies against ZIKV NS3 protein and by evaluating viral particle secretion at each time using RT-qPCR. In mosquito-transfected cells, no viral signal was detected at 1 day post-transfection (dpt). IFA positive was observed at 2 dpt, and the complete monolayer was antigen positive at 3 dpt for the WT virus (Fig. 4B), whereas no IFA signal was detected for the two mutants disrupting the SL1 structure. In contrast, disruption of stem 2 in SL2M1 mutant resulted in viruses that propagate like the WT ZIKV (Fig. 4B). To evaluate viral particle secretion in a quantitative manner, viral RNA for each mutant was quantified by RT-qPCR (Fig. 4B, right panel). The results indicate that the amount of secreted viral RNA of mutant SL2M1 was similar to that of the WT. In contrast, no secreted viral RNA was detected for the SL1-D and SL1-B mutants (Fig. 4B). In mammalian cells, replication of the ZIKV WT showed IFA positive at 1 dpt, with the complete monolayer infected at 2 dpt and cytopathic effect observed at 3 dpt (Fig. 4C). The two mutants in the SL1 structure showed delayed propagation with a few ZIKV antigen-positive cells at 1 dpt, but after 2 days, the monolayer was widely infected (Fig. 4C). In addition, the mutant SL2M1 propagated as efficiently as the WT. Quantification of the secreted viral RNA showed a delay of RNA accumulation for the mutants ZIKV SL1-D and SL1-B, but not for the SL2M1, compared with the WT ZIKV (Fig. 4C, right panel).

It is important to mention that although the nucleotide substitutions in SL1-D and SL1-B were synonymous, we cannot rule out an impact of those sequence changes on a viral function. However, since the mutants only carry one or two synonymous changes, and the phenotype recapitulates that observed in the reporter system (without nucleotide changes in the capsid coding sequence), we propose that the drastic impairment of viral replication in mosquito cells can be attributed to the disruption of the RNA structure.

**Fig 5:**
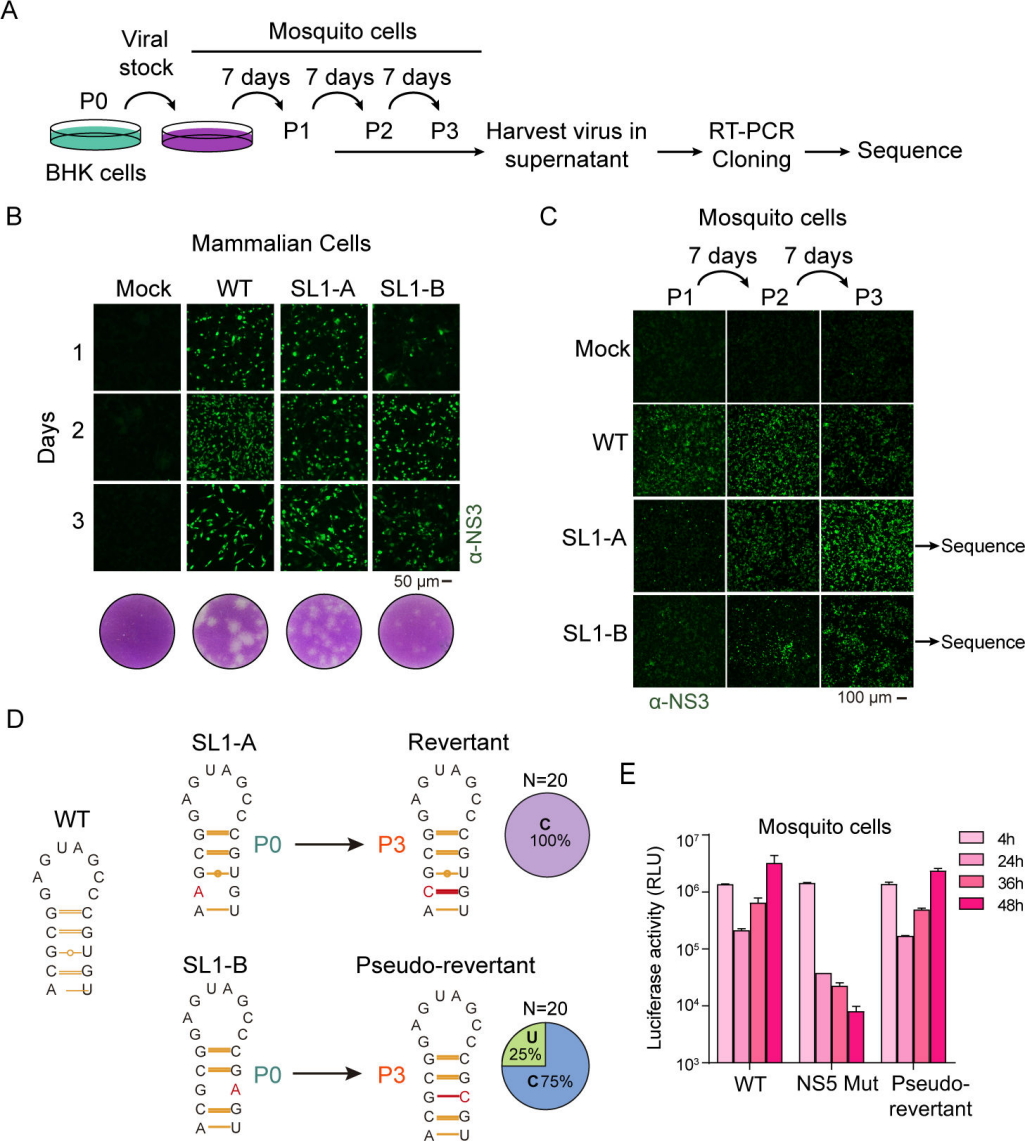
Mutations of SL1 disrupting the stem in a ZIKV infectious clone lead to revertant viruses in cell culture, restoring the structure. (**A**) Schematic representation of experimental procedure. Viral stocks of WT and mutant ZIKVs were prepared in mammalian cells (P0) and used to infect mosquito cells. The supernatant of this infection was used to infect fresh cells in serial passages, every 7 days. Three passages were obtained: P1, P2, and P3. P3 was harvested and used to rescue replicating viruses. Viral sequences were obtained by RT-PCR amplification and sequencing analysis. (**B**) Immunofluorescence assay images of transfected mammalian cells using a polyclonal anti-NS3 antibody. The replication and propagation of ZIKV WT, SL1-A, and SL1-B mutants are shown as a function of time post-transfection. Viral stocks were produced in these cells. On the bottom, representative images of the plaque phenotypes for each mutant. (**C**) Mutant ZIKVs gain replication ability in mosquito cells after serial passage. Immunofluorescence assay images of infected mosquito cells serially passaged every 7 days post-infection. A polyclonal anti-ZIKV NS3 antibody was used. (**D**) The selected viruses restore the SL1 structure. Schematic representation of SL1-A and SL1-B mutants in the initial stock (P0) and after three serial passages (P3). Revertants and pseudo-revertants were found at P3. The pie charts on the right show the proportion of clones obtained from each variant. (**E**) The identified pseudo-reversion restores viral replication. The mutation identified within SL1 was introduced into the ZIKV-Luc and used for the evaluation of RNA replication in comparison with the WT and the NS5 Mut control in mosquito cells. Luciferase levels are shown as a function of time (h: hours post-transfection). The luciferase values represent means ± standard deviations from one representative experiment of three biological replicates. Pairwise comparisons between WT and pseudorevertant virus at each time point were performed using an unpaired one-tailed Student’s *t*-test, and no significant differences were found.

The data support that the SL1 structure is critical for ZIKV replication in mosquito cells. To further investigate the importance of this RNA element, we tested infections of mosquito cells with viral stocks of mutants produced in mammalian cells. Viral stocks (~10⁷ pfu/mL) of WT, SL1-A, and SL1-B mutants were produced in BHK cells (Fig. 5A). The mutants exhibited small plaque phenotypes in these cells (Fig. 5B ), in agreement with their delayed RNA replication (Fig. 3 and 4). To rule out reversions or selection of additional mutations in mammalian cells, viruses were used to extract viral RNA for sequencing. This analysis confirmed that the original mutations were retained, and no additional changes were observed. Next, mosquito cells were infected with WT, SL1-A, or SL1-B, using a multiplicity of infection (MOI) of 1, and followed by successive passages of supernatants every 7 days (P1, P2, and P3) (Fig. 5A).

Viral replication was monitored by IFA (Fig. 5C). Mutant SL1-A showed signs of propagation at P2 (14 days), whereas mutant SL1-B propagated at P3 (21 days). To determine whether these mutants are associated with inefficient replication in mosquito cells or if adaptive mutations enhancing viral fitness were selected during passages, the harvested viruses were sequenced. Viral RNA from supernatants collected at P3 was extracted, amplified by RT-PCR, and the capsid-coding region was cloned and sequenced. Sequencing 20 clones revealed that SL1-A reverted to the WT sequence: the input mutation at position 162A reverted to 162C in all clones (Fig. 5D). Interestingly, sequencing of SL1-B clones revealed a pseudo-reversion in 15 out of 20 clones (75%), where the mutation 176A was replaced by 176C, creating a new CG base pair and restoring the stem. The remaining 25% of clones reverted to the WT sequence (176U, forming a UG base pair) (Fig. 5D). To confirm that the replicative phenotype observed was explained by the detected substitution and not by additional spontaneous mutations outside the capsid-coding region in the pseudo-revertant virus, the mutation 176C was introduced into the ZIKV-Luc construct and its ability to replicate in mosquito cells was evaluated. RNA from this mutant, along with WT and NS5 Mut controls, was transfected. The reporter ZIKV-RNA carrying the pseudo-reversion resulted in efficient viral RNA amplification in mosquito cells (Fig. 5E). The spontaneous emergence of a virus restoring the SL1 stem with an alternative sequence highlights a selective pressure to preserve this RNA structure, supporting its role in ZIKV replication in mosquito cells.

**Fig 6:**
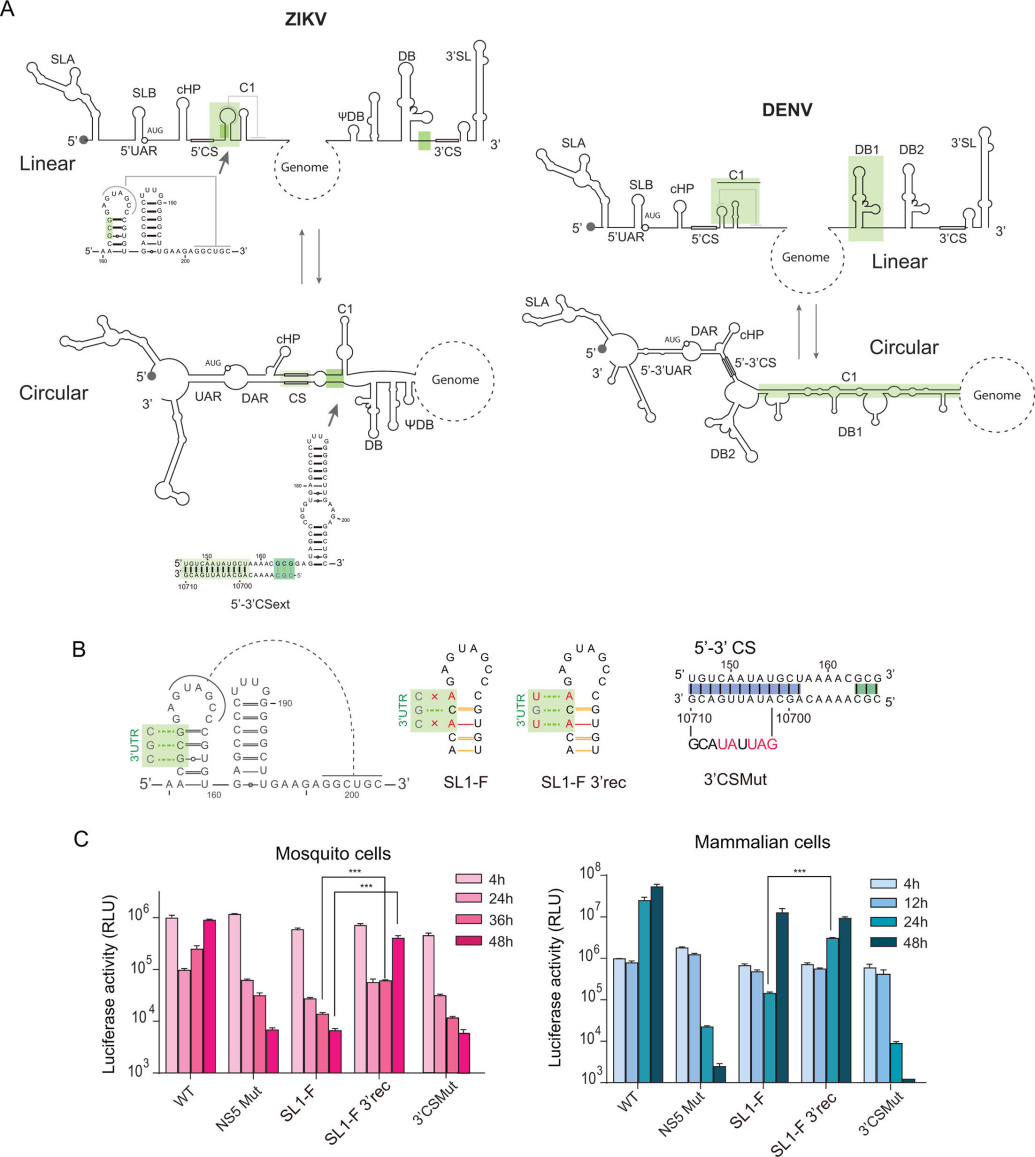
Long-range interaction extending CS1 is required for ZIKV replication. (**A**) Representation of circular and linear forms of the ZIKV and DENV genomes. Complementary regions are labeled (UAR, DAR, and CS), and cHP is indicated. In green, the involvement of C1 sequences is shown in different conformations. In the case of ZIKV, the nucleotide sequence of C1 in the stem loop and the hybridized 5’−3’ extended CS form, in the linear and circular conformations, is included. (**B**) Mutations that interfere with the formation of 5’−3’ extended CS with substitutions at the 5’ end SL1-F, and the complementary mutation at the 3’ end SL1-F 3’rec are shown. Also, a mutation that disrupts the 5’−3’CS, named 3’CS Mut, is included. (**C**) Luciferase expression levels of ZIKV-Luc WT and mutants are shown as a function of time (h: hours post-transfection). Left in mosquito cells and right in BHK cells. The luciferase expression shown corresponds to a representative experiment from three biological replicates. Graph displays the mean ± standard deviation (SD) of two technical replicates per condition. Pairwise comparisons between SL1-F and SL1-F 3’rec at each time were performed using an unpaired two-tailed Student’s *t*-test, as indicated. Differences were considered statistically significant at *P* values < 0.001 ***. Statistical analysis was performed using GraphPad Prism software.

To investigate the mechanism by which SL1 contributes to ZIKV replication, we compared its role with that previously reported for DENV (20, 35). In DENV2, most of the C1 sequence hybridizes with the DB1 sequence present at the 3’UTR (Fig. 6A), and this interaction was shown to promote genome cyclization and RNA synthesis. However, ZIKV 3’UTR lacks a DB1 structure (Fig. 6A). Analysis of the predicted circular and linear forms of the ZIKV genome suggests a potential interaction between SL1 sequence and nucleotides located upstream of CS1 at the 3’ end of the genome (Fig. 6A). This long-range interaction requires unfolding of SL1, extending the known 5’−3’ CS1 interaction by three base pairs.

To assess the relevance of this potential long-range interaction, we designed a ZIKV mutant (SL1-F) with substitutions in SL1 disrupting the predicted complementarity with the 3’ end, without affecting SL1 structure. Complementarity was restored in a separate mutant (SL1-F 3’rec) by introducing compensatory mutations at the 3’ end of the genome. An additional mutant was included (3’CSMut), carrying a substitution in the 3’CS1 sequence, without affecting C1 (Fig. 6B). The mutated viral RNAs (SL1-F, SL1-F3’rec, and 3’CSMut) were transfected into mosquito and mammalian cells, alongside the WT and the NS5-Mut as controls.

The mutation disrupting CS1 (3’CSMut) impaired replication in both hosts, supporting the essential role of this element in genome cyclization (Fig. 6C). The SL1-F mutation, which disrupts the formation of the extended CS1 interaction, impaired replication in mosquito cells but caused only a delay in replication in BHK cells. Notably, the mutant RNA, in which complementarity was restored by substituting 3’ end nucleotides (SL1-F3’rec), achieved approximately 100-fold higher RNA amplification level compared with SL1-F in mosquito cells. In mammalian cells, this mutant exhibited a slight improvement in replication (Fig. 6C). These findings support a role of the extended 5’−3’ CS for viral replication in a host-dependent manner.

## DISCUSSION

Here, we search for functional RNA structures present in the coding sequence of the ZIKV genome. Our data show that the first 100 nucleotides of the capsid-coding sequence contain cis-acting RNA elements for viral replication. This information is relevant for constructing reporter systems and for designing attenuated viruses. Specifically, we identified an RNA stem-loop structure, SL1, present in the coding sequence that is important for viral RNA replication in a host-specific manner. The integrity of SL1 was found to be crucial for ZIKV infectivity, and disrupting the stem structure was sufficient to abolish viral replication in mosquito cells. The SL1 structure is formed in the linear conformation of the genome, but its sequence also hybridizes with a sequence upstream of the conserved 3’CS1 at the 3’UTR, generating a long-range interaction. It is important to mention that disruption of the stem of SL1 without altering complementarity with the 3’ end of the genome impairs ZIKV replication, supporting a role of the SL1 structure in the linear form of the genome. Our data suggest that both RNA structures, SL1 and 5’−3’ extended CS, are necessary for viral RNA replication, supporting dynamic conformations of the ZIKV genome during infection.

The SL1 structure is part of a larger conserved RNA element originally described in DENV as DCS-PK or C1 (20, 35). This element contains several structures: SL1, SL2, and a pseudoknot interaction (PK). For clarity, the previously reported structures using DENV4 and DENV2 are included in the supplemental material. A structure homologous to SL2 of DENV and ZIKV was recently described to be relevant for YFV replication (40). In the context of DENV4, the conserved DCS-PK structure was systematically studied, and it was reported that mutations altering the PK and the stems of SL1 and SL2 reduce viral replication in mammalian cells, whereas this was not assessed in mosquito cells. We have previously reported that the sequence of C1 was necessary for optimal DENV2 replication, and that nucleotides of this structure hybridize with the sequence of the conserved DB1 structure at the viral 3’UTR (13, 35). Here, we present data that supports substantial differences in the role of C1 in ZIKV and DENV. First, the SL1 structure was found to be essential for ZIKV infection in mosquito cells. The crucial role of SL1 was supported by using reporter viruses and infectious clones with mutations that disrupted the stem of SL1 or restored the structure by artificial mutations or by naturally emerging reversions (Fig. 3 to 5). Second, using ZIKV with mutations disrupting SL2 or the PK interaction, we showed that these elements were dispensable for ZIKV RNA replication in both hosts (Fig. 2). Even the deletion of the complete SL2 showed a minor effect on viral RNA replication, in contrast to that shown for DENV (20, 35). Third, a sequence within SL1 was found to be involved in a long-range interaction with a sequence at the 3’ end of the genome located upstream of 3’CS, enhancing viral RNA replication, which also differs from that previously reported for DENV (Fig. 6).

It is noteworthy that the closely related DENV and ZIKV share a conserved RNA structure within the capsid-coding sequence that modulates genome cyclization, but through the interaction with different regions of the 3’ UTR. We speculate that the topology of the RNA around the 5’ end of the genome is critical because it is near the RNA promoter for polymerase binding (3), which is conserved in all flaviviruses. The data suggest that the location of complementary sequences at the 3’ end is less critical. From the mosquito-borne flavivirus groups (MBFV), the members of the Spondweni (including ZIKV) and the YFV groups contain a single DB structure at the 3’UTR, whereas a conserved duplication of the DB structure is found in most of the other MBFV (37). It is important to mention that the two DB structures in the DENV genome are not redundant; instead, they play different functions during viral replication (13). It is possible that ZIKV lost one copy of the DB structure, forcing evolution of alternative sites for cyclization at the 3’ end of the genome. In agreement with the idea of diversification of regulatory mechanisms for genome cyclization among MBFV, a recent report highlights differential requirements for YFV. In this case, a regulatory RNA element at the coding sequence of the capsid, homologous to the SL2 of DENV and ZIKV, assists a balanced equilibrium between linear and circular forms of the YFV genome (40), a process that has been previously shown to be critical for DENV infectivity (11).

Our studies, together with findings from other groups, have demonstrated that disrupting cyclization elements results in a more severe impact on flavivirus replication in mosquito cells than in human cells. For example, in the case of DENV, disruption of complementarity between UAR and DAR elements demonstrated that single mismatches impaired replication in mosquito cells without significantly affecting replication in mammalian cells (38). Similarly, disruption of the C1-DB1 interaction has been reported to have a differential impact on viral replication, with a greater effect observed in mosquito than in mammalian cells (35). In the case of ZIKV, disruption of the complementary DAR sequence was also shown to have minimal impact on viral replication in mammalian cells but caused a dramatic impairment in viral propagation in mosquito cells (29). Consistent with these findings, we observed here that alterations in the 5'−3' extended CS complementary region had a critical effect on ZIKV replication in mosquito cells, whereas the impact on replication in mammalian cells was relatively minor. These observations suggest that the equilibrium between the linear and circular conformations of the viral genome displays different requirements in the two hosts, with a stronger constraint in insect cells. This may be influenced by factors such as the distinct temperatures at which the virus replicates in the two hosts or differences in host proteins that interact with the viral RNA.

The conserved location of functional RNA elements in flavivirus capsid-coding sequences is intriguing. A selective pressure to position critical RNA elements near the 5’ end of the genome may have shaped the capsid-coding sequence to be more adaptable, allowing it to accommodate these essential regulatory structures without compromising protein function. In this regard, the capsid protein is the least conserved of the flavivirus proteins, likely reflecting an evolutionary tolerance for variability in this region. Alignments of capsid-coding sequences from different flaviviruses show less than 40% sequence identity (41). For DENV, a large flexibility in the amino acid sequence was found in the N-terminal of the capsid, provided that the nucleotide sequence of the cis-acting elements was conserved (42). A remarkable flexibility in the capsid protein of TBEV was also found, which tolerated large deletions ranging from 19 to 30 residues (43). The related avian flavivirus, tembusu virus, is another example, where large deletions of capsid spanning almost the entire C-terminal helix were tolerated, and infectious particles were produced (44). This coding sequence flexibility could be associated with the need to accommodate cis-RNA sequence/structure requirements.

In summary, we describe a functional RNA element in the capsid-coding sequence of the ZIKV genome with distinct requirements in the two hosts. A detailed understanding of relevant RNA signals in the viral genome of important human pathogens will help design genetic tools for rational antiviral strategies.

## MATERIALS AND METHODS

### Cell culture

Baby hamster kidney cells (BHK-21) were cultured in Minimum Essential Medium α (MEMα) (Gibco, Thermo Fisher Scientific) supplemented with 10% fetal bovine serum (FBS) (Gibco, Thermo Fisher Scientific) and 100 U/mL penicillin-streptomycin in 100 mm cell culture plates and incubated at 37°C with 5% CO2. Huh-7 cells (human hepatocyte cell line) were cultured in Dulbecco’s modified Eagle’s high-glucose medium (4,500 mg/L) supplemented with 10% fetal bovine serum, 100 U/mL penicillin, and 100 µg/mL streptomycin. Mosquito C6/36 HT cells were cultured in Leibovitz’s L-15 Medium (Gibco, Thermo Fisher Scientific) supplemented with 10% FBS, 100 U/mL penicillin-streptomycin, 0.3% tryptose phosphate broth, 0.02% glutamine, 1% minimal essential medium (MEM) nonessential amino acids solution, and 0.25 µg/mL amphotericin B (amphotericin B) in T75 cell culture flasks, and incubated at 33°C.

### Construction of recombinant Zika viruses

For constructing the recombinant full-length ZIKV luciferase reporter containing mutations in the capsid-coding region, we modified a ZIKV reporter infectious clone that we have previously described (36). For every mutant, we replaced the MluI-XhoI fragment of the clone with a PCR product obtained by overlapping PCR, generated using primers containing the desired mutations. For the non-reporter mutants, we employed the same overlapping PCR methodology and replaced the MluI-AvrII fragment of the ZIKV full-length infectious clone previously reported (36). The ligation products were transformed into XL1-Blue bacteria, and several clones for each mutant were obtained. The resulting plasmids were sequenced, and the positive clones were used for *in vitro* RNA transcription. The sequence of the oligonucleotides used is included in Table S1.

### RNA transcription and transfection

WT or recombinant ZIKV infectious clone plasmids were linearized by digestion with KpnI and used as templates for *in vitro* transcription by Ambion T7 RNA Polymerase (Ambion, Thermo Fisher Scientific) in the presence of m7GpppA cap structure analog (New England Biolabs), and incubated for 2 h at 37°C. RNA integrity was analyzed by agarose gel electrophoresis, and the concentration was measured using Qubit RNA HS Assay Kits (Thermo Fisher Scientific).

RNA transfections were performed using Lipofectamine 2000 (Thermo Fisher Scientific) and Opti-MEM medium (Gibco) according to the manufacturer’s instructions. For 24-well cell culture plates, 100 ng of viral RNA per well was used. Renilla luciferase assays were performed using the Renilla luciferase assay system kit (Promega).

### Immunofluorescence assay

BHK-21 or C6/36 HT cells were grown in 24-well cell culture plates containing 12 mm glass coverslips. At the corresponding times post-transfection or infection, the coverslips were collected, and the cells were fixed with methanol for 15 min at –20°C. The coverslips were blocked using gelatin 0.2% (Sigma) in PBS and incubated with rabbit anti-NS3 polyclonal antibody diluted 1/500 in blocking solution. Goat anti-rabbit antibody Alexa Fluor 488 conjugate (Thermo Fisher Scientific) was employed to detect the primary antibody, and DAPI (Thermo Fisher Scientific) was added to visualize the nuclei. The counterstained nuclei of the immunofluorescence shown in Fig. 4 and 5 are included in Fig. S3 and S4, respectively. Images were obtained using an Axio Observer 3 (Zeiss) inverted fluorescence microscope, with 10× and 20× objectives.

### Viral infections and plaque assays

C6/36 HT cells were seeded in 24-well cell culture plates and grown overnight. Transfected BHK-21 cells supernatants were used to infect C6/36 HT monolayers for 1 h at 33°C. Then, the inoculum was removed, and 500 µL of Leibovitz’s L-15 Medium supplemented with 5% FBS was added to each well. For serial passages, 200 µL of infection supernatants were employed to infect new C6/36 monolayers.

For viral plaque assays, BHK-21 cells were seeded in 24-well cell culture plates and grown overnight. Transfected BHK-21 cells supernatants were serially diluted, and 200 µL of the inoculum was used to infect BHK-21 monolayers for 1 h at 37°C. Afterward, 1 mL of overlay medium (MEMα, 0.8% methyl cellulose, supplemented with 5% FBS) was added to each well. Cells were fixed 7 days post-infection with 10% formaldehyde and stained with crystal violet.

### RNA secondary structure prediction

RNA secondary structures of WT and mutants were predicted using two independent models: IPKnot (45) and AlphaFold 3 (46). IPKnot is a model that accounts for secondary structures, including pseudoknots. In the case of Alphafold 3 predictions, secondary structures were extracted from the three-dimensional models using RNApdbee 3.0 (47). Visualization of three-dimensional structures was performed using ChimeraX v1.10.1 (48), and FORNA (49) was used for visualizing secondary structures.

### RNA extraction and quantification

For qRT-PCR of viral RNA, TRIzol reagent (Invitrogen) was used for RNA extraction of supernatants. cDNA was synthesized with random decaoligonucleotide primer mix by M-MLV Reverse Transcriptase (Promega) as previously described (36). qPCRs were performed in duplicates in 96-well plates using 2 µL of the RT reaction mixture as template, 5 µL of FastStart SYBR green Master 2 x mix (Roche), a 600 nM concentration of each primer, and RNase-free water up to 10 µL. The primers AVG2098 (5′-GCCGCCACCAAGATGAACTGATTG-3′) and AVG2099 (5′-GCAGTCTCCCGGATGCTCCATC-3′) were targeted to amplify nucleotides 9,854 to 9,927 within the NS5 coding sequence.

## Data Availability

All data supporting the findings of this study are available within the paper and its supplemental material.

## SUPPLEMENTAL MATERIAL

Supplemental material jvi.01550-25-s0001.pdf Figures S1 to S4, schematic representations of structures, and Table S1.

ASM does not own the copyrights to Supplemental Material that may be linked to, or accessed through, an article. The authors have granted ASM a non-exclusive, world-wide license to publish the Supplemental Material files. Please contact the corresponding author directly for reuse.
