# Supplementary material for: A conserved RNA structure at the capsid-coding sequence of Zika virus genome is required for viral replication in a host-dependent manner
Source: J Virol. 2025 Oct 13;99(11):e01550-25. doi: 10.1128/jvi.01550-25 (PMC12645915; doi:10.1128/jvi.01550-25)
Supplement: Supplemental material — Figures S1 to S4, schematic representations of structures, and Table S1. [file jvi.01550-25-s0001.pdf]

Figure S1

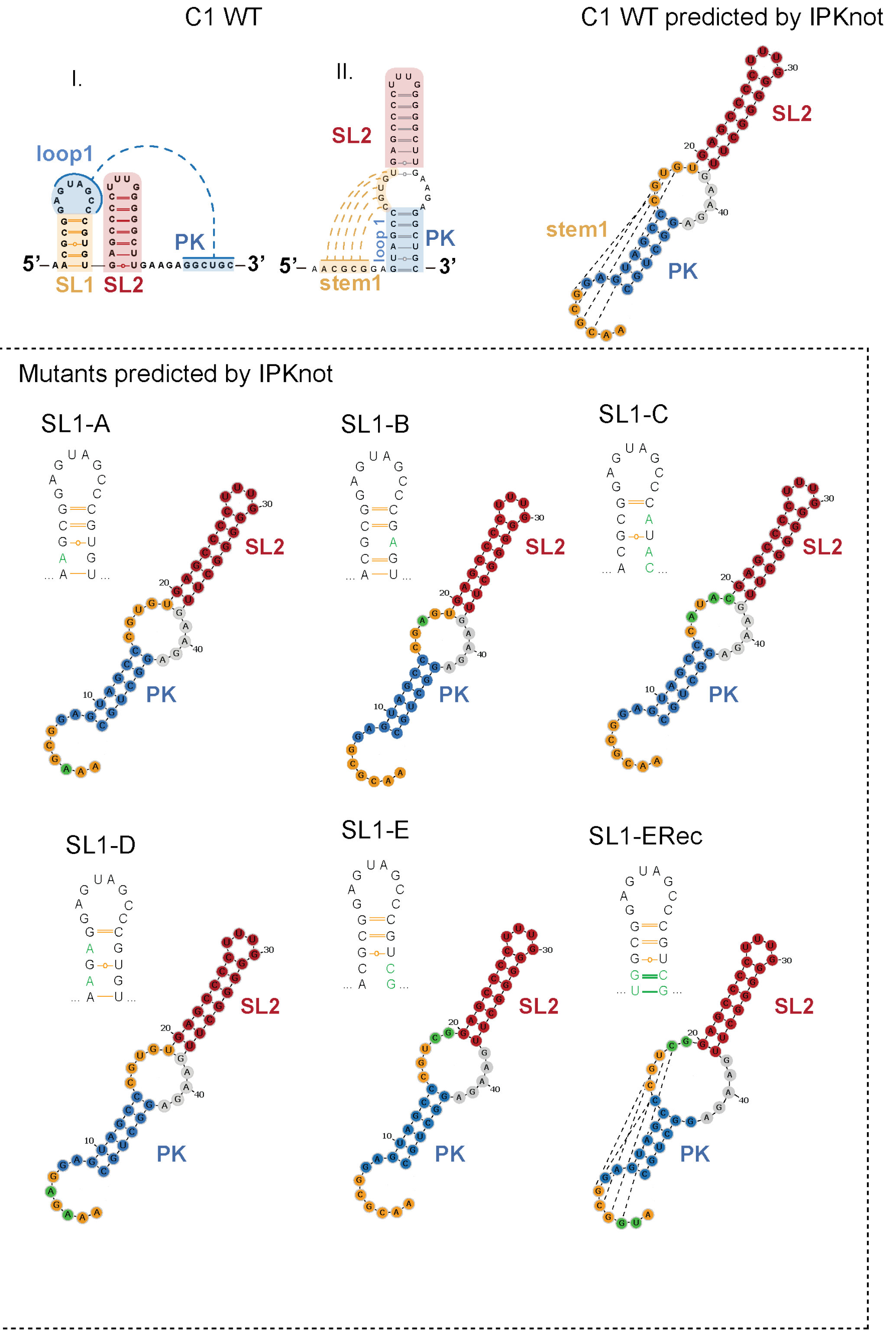

Figure S1. Prediction of the C1 structure WT and SL1 mutants. On the top, there are two alternative representations of the structure based on chemical probing and prediction using IPKnot algorithm, as indicated. On the bottom, prediction of SL1 mutants using IPKnot. The nucleotides corresponding to substructures SL1, SL2 and PK are color coded.

Figura S2

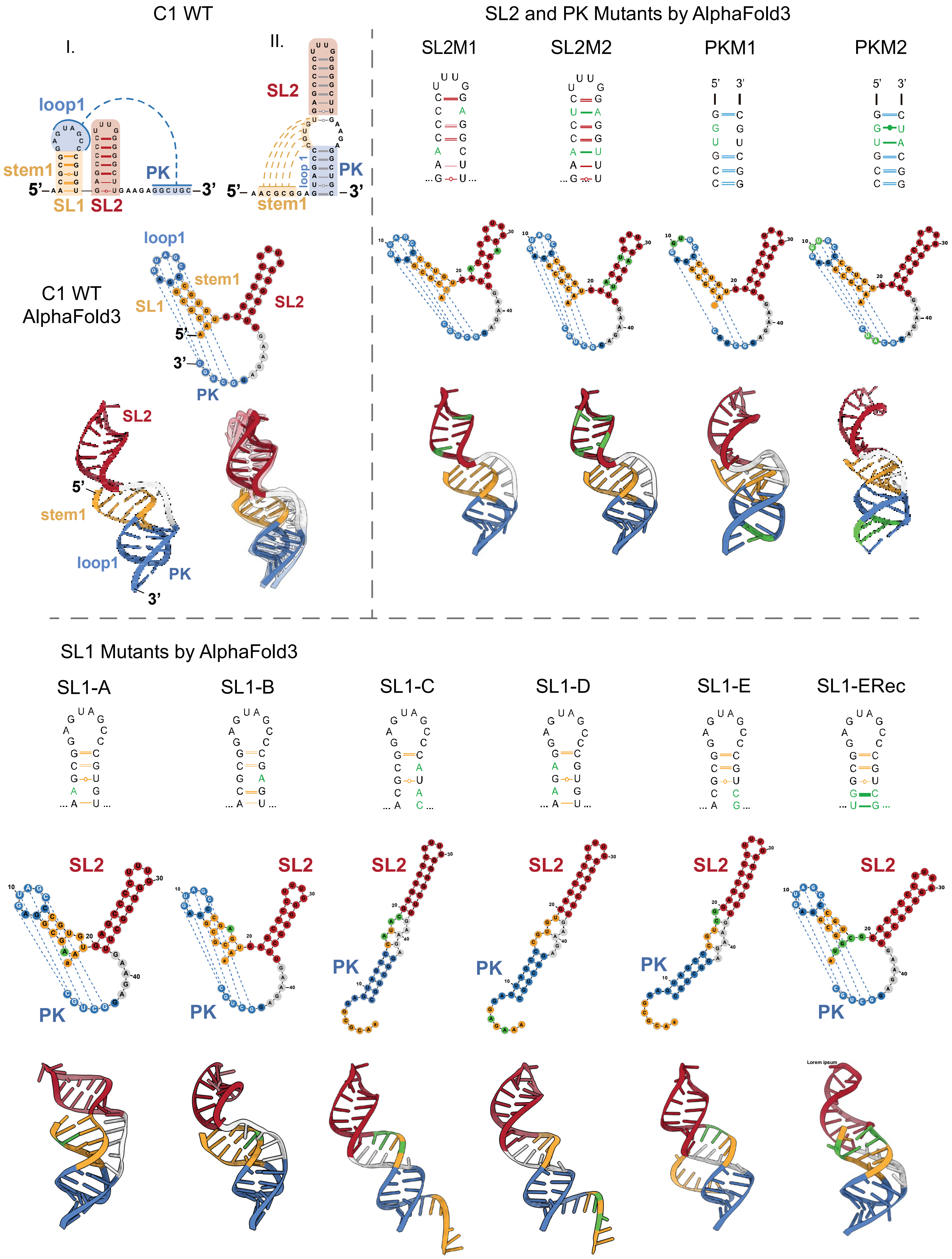

Figure S2. Prediction of the C1 structure WT and mutants using AlphaFold3. The 3D structures and the 2D projections are shown for the WT and each mutant as indicated in each case. The nucleotides corresponding to substructures SL1, SL2 and PK are color coded.

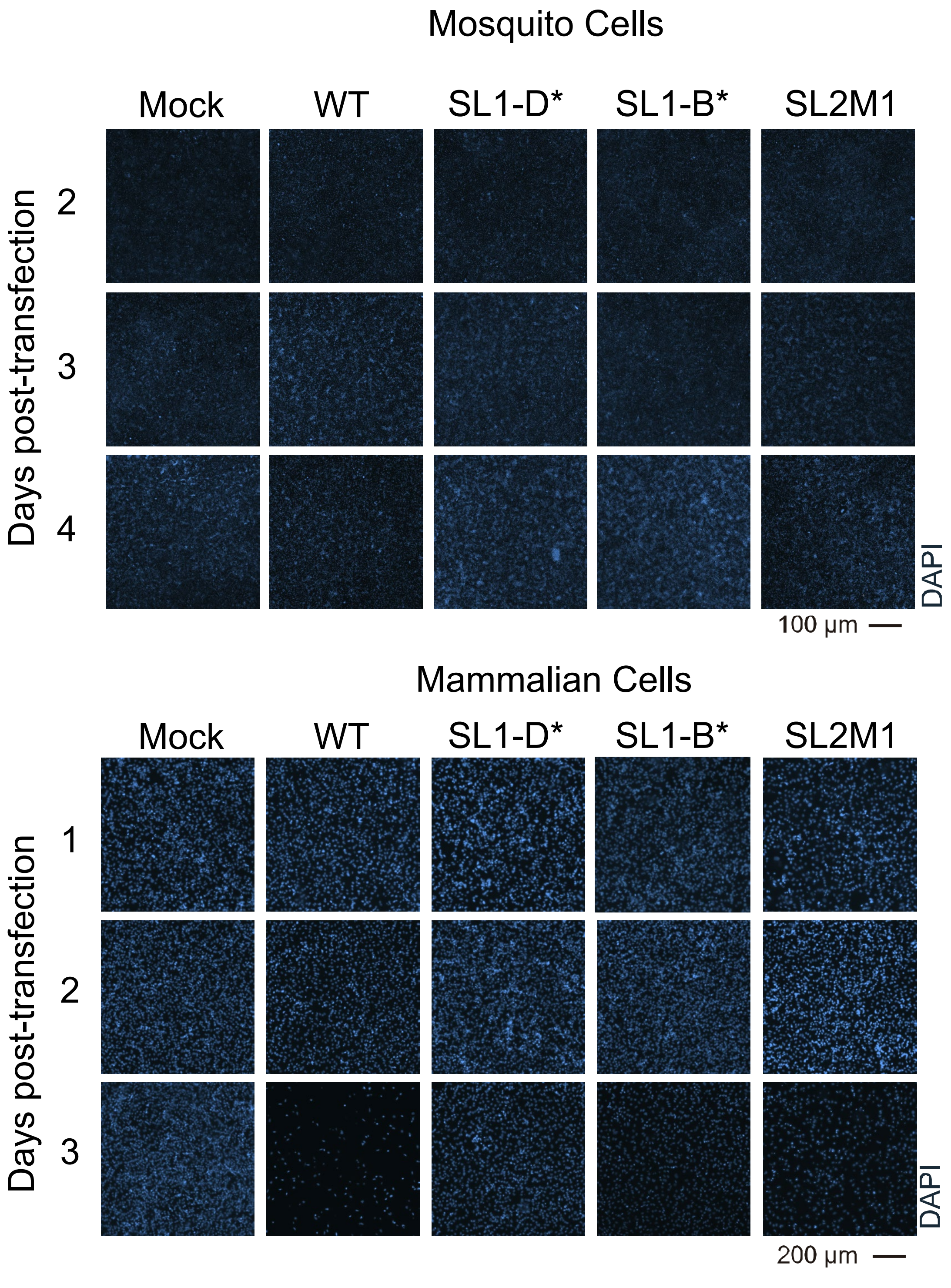

Figure S3. DAPI counterstaining of immunofluorescence images shown in Figure 4

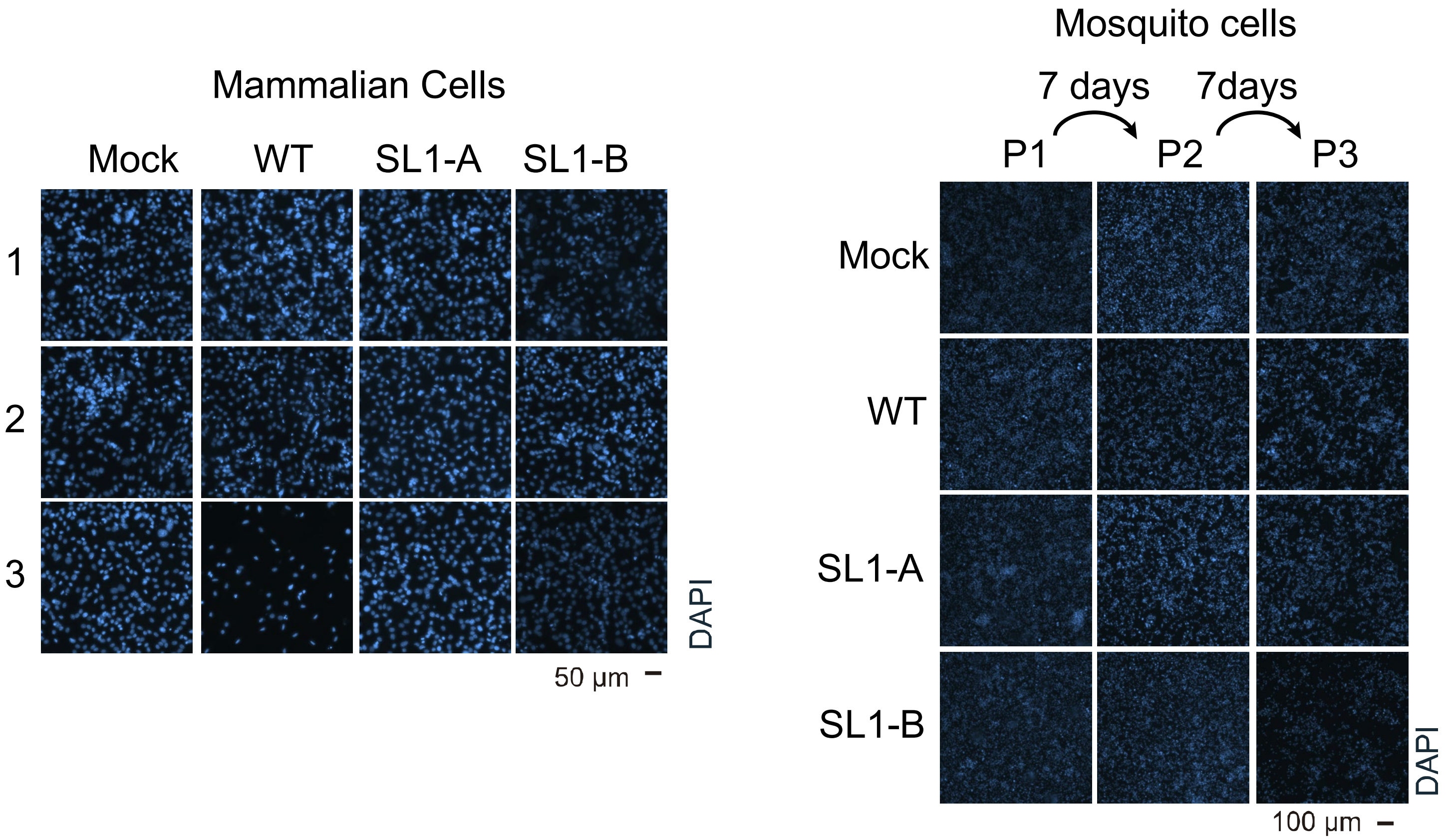

Figure S4. DAPI counterstaining of immunofluorescence images shown in Figure 5

Supplemental material: Schematic representations of the structure DCS-PK as described in DENV4 genome Liu et al *JVI*, 2013

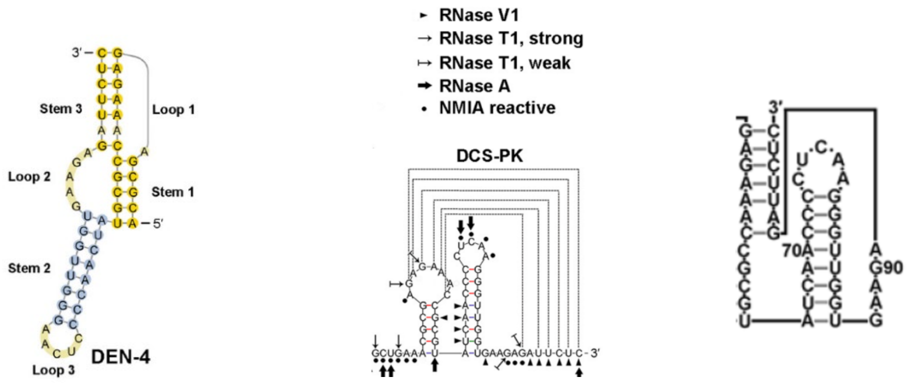

Structure of C1 as described in DENV2, de Borba et al *JVI*, 2015, showing the SHAPE reactivity in the linear and circular forms of the RNA. On the right, nucleotide sequence of the C1 structure of DENV2.

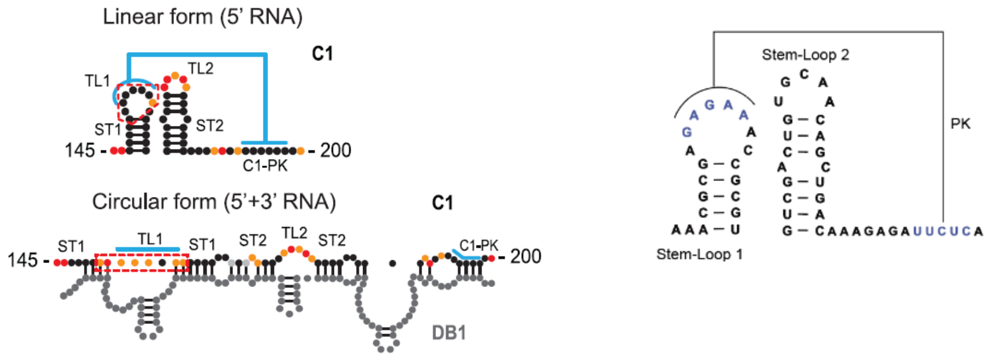

Schematic representation of the circular form of the DENV2 genome, including structures at the 5' and 3' end of the genome (de Borba et al *JVI*, 2015)

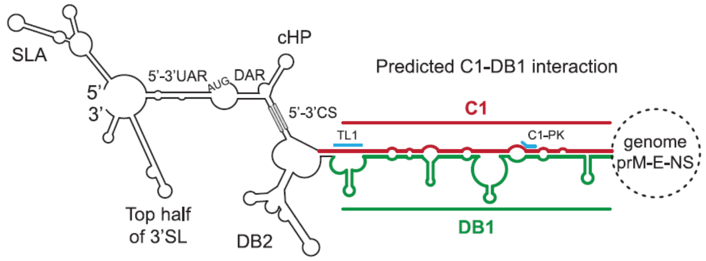

Schematic representation of DCS-PK structure of ZIKV genome as defined by chemical probing (Pan Li et al. 2018, *Cell Host and Microbe*)

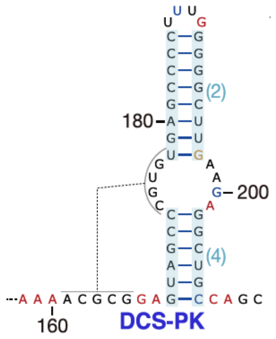

Supplementary Table: sequence of oligonucleotides used in the manuscript

Oligonucleotide Sequence	Name of mutant
GGCAGTCAGTGcAGcTGcTTGCGTTGTGAAGCCAATTGATGATAGG	NS5 Mut For
CGCAAgCAgCTgCACTGACTGCCATTCGTTTGAGCC	NS5 Mut Rev
CAGAAGTCCGGCTTTTAGCATATTGACAATCCGGAATCCTCC	ΔC1 Rev
CAATATGCTAAAAGCCGGACTTCTGCTGGGTCATGGG	ΔC1 For
AAAGGCTAGAATTGGCAGCCTCTTCAAGCCCCC	ΔK Rev
AAGAGGCTGCCAATTCTAGCCTTTTTGAGATTCACGGC	ΔK For
CTTTATTATTTCCAGAATCGCCAAGACCATCCTGATGGG	ΔL Rev
GTCTTGGCGATTCTGGAAATAATAAAGAAGTTCAAGAAAGATCTGGC	ΔL For
aggagctgCttaagTCTTTCCATAGCCTCTTTTTTCCCCACT	ΔM Rev
AGAGGCTATGGAAAGActtaaGcagctcctaaattttgatttactcaaac	ΔM For
CCCGTGTGAaCCCCTTTGGaGGCTTGAAGAGGC	SL2 M1 For
GCCTCTTCAAGCCtCCAAAGGGGtTCACACGGG	SL2 M1 Rev
AGCCCGTGTGAaCCtCTTTGGaGGtTTGAAGAGGCTGCC	SL2 M2 For
GGCAGCCTCTTCAAaCCtCCAAAGaGGtTCACACGGGCT	SL2 M2 Rev
GTAGCCCGTGTGAAGAGGCTGCCAGCCGGACTTCTG	ΔSL2 For
GGCAGCCTCTTCACACGGGCTACTCCGCGTTTTAGCA	ΔSL2 Rev
CGCGGAGgtGCCCGTGTGAGCCCC	PK M1 For
GGCTCACACGGGCacCTCCGCGTTTTAGC	PK M1 Rev
CGCGGAGgtGCCCGTGTGAGCCCCTTTGGGGGCTTGAAGAGGCatCCAGCCGGAC	PK M2 For
TTTTAGC	PK M2 Rev
AATATGCTAAAAaGCGGAGTAGCCCGTG	SL1-A For
CGGGCTACTCCGCtTTTTAGCATATTGAC	SL1-A Rev
CGCGGAGTAGCCCGAGTGAGCCCCTTTGGGG	SL1-B For
CCCCAAAGGGGCTCACTCGGGCTACTCCGCG	SL1-B Rev
GAGTAGCCCatacGAGCCCCTTTGGGGGCTTG	SL1-C For
GCCCCCAAAGGGGCTCgtatGGGCTACTCCGC	SL1-C Rev
GTCAATATGCTAAAAaGaGGAGTAGCCCGTGTGAG	SL1-D For
CTCACACGGGCTACTCCtCtTTTTAGCATATTGACAATCC	SL1-D Rev
GCTAAAACGCGGAGTAGCCCGTcgGAGCCCCTTTGGGGG	SL1-E For
CCCCCAAAGGGGCTCcgACGGGCTACTCCGCGTTTTAGC	SL1-E Rev
GCTAAAtgGCGGAGTAGCCCGTcgGAGCCCCTTTGGGGGC	SL1-E Rec For
GCCCCCAAAGGGGCTCcgACGGGCTACTCCGCcaTTTAGCATATTG	SL1-E Rec Rev
CGCGGAGTAGCCCGcGTGAGCCCCTTTGGGG	Pseudo-Revertant For
CCCCAAAGGGGCTCACgCGGGCTACTCCGCG	Pseudo-Revertant Rev
GTCAATATGCTAAAACaCaGAGTAGCCCGTGTGAGCC	SL1-F For
TCACACGGGCTACTCtGtGTTTTAGCATATTGAC	SL1-F Rev
CCCCCCGGAAAAtGtAAAACAGCATATTGAC	SL1-F 3' Rec For
TATGCTGTTTTaCaTTTTCCGGGGGGTCTCC	SL1-F 3' Rec Rev
CCCGGAAAACGCAAAACAGCGATTATACGCTGGGAAAGACC	3'CS Mut For
GGTCTTTCCCAGCGTATAATCGCTGTTTTGCGTTTTCCGGG	3'CS Mut Rev
